# Association between the utilization of senior centers and participation in health check-ups

**DOI:** 10.1038/s41598-024-61995-3

**Published:** 2024-05-21

**Authors:** Ah Jung Ko, Jinhyun Kim, Eun-Cheol Park, Min Jin Ha

**Affiliations:** 1 Department of Health Policy & Management, Graduate School of Public Health, Yonsei University, Seoul, Republic of Korea; 2 Institute of Health Services Research, Yonsei University, Seoul, Republic of Korea; 3 Department of Preventive Medicine, Yonsei University College of Medicine, Seoul, Republic of Korea; 4 Department of Psychiatry, Yonsei University Hospital, Seoul, Republic of Korea; 5 Department of Health Informatics and Biostatistics, Graduate School of Public Health, Yonsei University, 50 Yonsei-ro, Seodaemun-gu, Seoul, 03722 Republic of Korea

**Keywords:** Health check-ups, Senior center, Older adults, Social interaction, Health behavior, Health care, Risk factors

## Abstract

The global older adult population is increasing. Early detection and intervention through health check-ups are crucial for successful aging, as they play a significant role in identifying and addressing diseases. This study explored the relationship between the utilization of senior centers and the promotion of health check-ups. It utilized data from 10,097 individuals aged 65 years and above, sourced from the 2020 Elderly Survey in South Korea. The primary variable of interest was classified into two groups: those who utilized senior centers and those who did not. Subgroups were further categorized based on the frequency of usage and the presence of family members among senior centers users. Logistic regression analyses were conducted to assess the association between the utilization of senior centers and participation in health check-ups. Both men and women utilizing senior centers demonstrated a higher likelihood of participating in health check-ups compared with those who did not use senior centers. Participants visiting senior centers in a week exhibited a progressively higher likelihood of engaging in health check-ups compared with those who visited such senior centers zero times a week. Senior centers can serve as effective intervention methods to enhance health check-ups among older adults. Furthermore, this can contribute to fostering successful aging among older adults.

## Introduction

The utilization of health check-ups among older adults contributes to the early detection of diseases, thereby reducing mortality rates and enhancing quality of life^1^. Timely intervention resulting from early disease detection can slow the progression illnesses and decrease medical expenditures for treatment^2^. As aging advances, individuals become more susceptible to diseases, necessitating active management of both physical and mental aspects^3,4^. The global aging population and increased life expectancy have contributed to a rising incidence of chronic non-communicable diseases (NCDs) and multimorbidity^5^. These chronic NCDs often diminish the quality of life of older adults and can serve as a leading cause of death^6^. Through health check-ups, older adults become aware of their existing diseases and risk factors early on, enabling the postponement of diseases and providing an opportunity to receive treatment^6,7^. Thus, regular health check-ups prove to be an effective method for disease prevention and maintaining health in successful aging^8^. Health check-ups offer the advantage of gaining insight into one’s own health status, thereby increasing the likelihood of adopting healthy behaviors^9^. In other studies, it has been noted that older adults who undergo annual health check-ups have a higher probability of survival^10^. Research indicates that regular health check-ups contribute to early detection and the extension of lifespan by identifying health risk factors such as total cholesterol and body mass index^11,12^. Particularly, chronic conditions like hypertension and diabetes necessitate consistent monitoring^13^.

Andersen’s healthcare utilization model posits that healthcare utilization is influenced by four factors beyond environmental, demographic characteristics, and health behaviors, extending to health outcomes^14^. The value attributed to health, alongside other motivational factors, demographic characteristics, collectively influences the inclination to engage in health check-up behaviors^15^. Among older adults, harboring negative health beliefs, lacking encouragement for health from their surroundings, or facing economic hardship is associated with a decreased likelihood of participating in health check-ups^16^. Health literacy plays a crucial role in promoting engagement in health check-ups^17^. However, older adults often exhibit lower health literacy than younger individuals, posing challenges in acquiring health knowledge^17^. For instance, the prevalence of depression among older adults is notably high^18^. In South Korea the incidence of depression among older adults ranges from 13.87 to 18.36%, with a tendency to increase with age^19^. Older adults experiencing geriatric depression are less likely to adopt health-promoting behaviors compared with those not experiencing it^20^. Furthermore, the post-retirement experience of anxiety contributes to this mentally vulnerable state, hindering the utilization of health check-ups among older adults^21,22^. On the other hand, social support serves as motivating factors for older adults to engage in healthy behaviors^23^. Through participation in social activities, older adults can naturally engage in healthy behaviors, and those with social connections tend to place a higher value on health compared to those without^24,25^. Participation in senior centers is part of social interaction. The characteristics of older adults utilizing senior centers include being female, having higher income, and having good accessibility to senior centers. Additionally, older age is associated with higher attendance rates at senior centers^26,27^.

According to previous research, individuals from larger families and those with high social engagement and extensive connections within their local community, are more inclined to participate in health check-ups. This underscores the positive influence of interactions among family members and neighbors on health behaviors^8^. Another study highlighted that individuals who perceive a lack of support from society are more likely to forgo health check-ups^22,28–30^. Earlier research has indicated that social relationships were identified as factors associated with participation in health check-ups for both men and women^31,32^. However, there is a scarcity of studies focusing on whether social activities within peer groups affect participation in health check-ups. Based on the author's findings, there is no existing research on the association between the use of community centers by the elderly and health screenings. As part of older adult community participation, senior centers serve as communal spaces where older adults can engage in various activities with their peers. Within these centers, individuals receive education on health and digital-related topics, share meals, and exchange cultural experiences. This study aims to explore the association between engaging in social activities with peers within senior centers and participation in health check-ups.

## Methods

### Data and study population

This study utilized data from the 2020 Elderly Survey conducted by the Ministry of Health and Welfare and the Korea Institute for Health and Social Affairs^33^. The Korean Elderly Survey investigates the living conditions and welfare needs of individuals aged 65 years and above in South Korea^34^. The sampling design employs a two-stage stratified sampling, with the first stage stratifying by survey districts and the second stage by surveyed households^35^. The survey is conducted through in-home interviews^33^. Participants provide informed consent before the survey commences. Additionally, the survey data are publicly accessible, eliminating the need for ethical approval^34^. The survey was conducted from September 14 to November 20, 2020. The survey was conducted on 10,097 people (4035 men and 6062 women) over the age of 65 living in 17 districts. This study was conducted during the COVID-19 period in 2020. The COVID-19 period has distinct characteristics such as reduced social activities, which may introduce other potential impacts on the research results.

### Variables

The dependent variable, participation in health check-ups, was categorized according to responses to two questions: “Have you undergone any health check-ups, excluding dementia screenings, in the past two years?” and “Have you undergone dementia screenings in the past two years?” Participants responding “yes” to both questions were categorized as having participated in health check-ups, whereas those participating in only one of the health check-ups or not participating in both were classified as non-participants. Health check-ups refer to general health check-ups and other health check-ups conducted by government agencies^36^. General health check-ups aim to identify manageable conditions such as hypertension, diabetes, depression, and hepatitis through early detection^37^. Dementia screenings are conducted targeting individuals aged 60 and above to detect cognitive impairment early. Screenings can be scheduled at public health centers or designated hospitals affiliated with the government at the individual’s preferred time^38^.

The primary variable of interest in this study, the utilization of senior centers, was assessed by asking individuals whether they had utilized senior centers in the past year. Senior centers include local centers, elderly welfare centers, social welfare centers, women’s support centers, senior citizen classrooms, public leisure, and cultural facilities. These centers refer to facilities operated by both public and private entities targeting the elderly. Various programs such as education, hobbies, social activities, and meal services are conducted in these centers. Participants’ responses were recorded as “yes” or “no.” Additionally, participants who reported utilizing senior centers were classified into three subgroups based on the frequency of utilization (more than five times a week, 3–4 times a week, 1–2 times a week) and family composition (living with family, living alone).

We considered sociodemographic, physical, and mental health–related factors as potential confounding variables and controlled for them. Sociodemographic factors comprised sex, education level (≤ middle school, high school, ≥ college), income (low, middle, high), Job status (yes, no), and family interaction (low, middle, high). Family interaction was categorized based on the frequency of meeting family members, such as children and relatives, into high, middle, and low categories. The family interaction variable was classified as high if there was frequency of meeting on a weekly basis, moderate if it occurred every 1–3 months, and low if it occurred on a yearly basis. Physical and mental health-related factors encompassed nutritional status (low, middle, high), alcohol status (yes, no), smoking status (yes, no), physical function (yes, no), physical activity (yes, no), subjective health status (low, middle, high), and depression (normal, moderate, severe). The subjective health status was assessed by asking participants how they perceived their own health status. Responses were categorized as very healthy, healthy (high), average (moderate), poor, or very poor (low). Nutritional status was assessed utilizing the tool developed by the nutrition screening initiative, while depression was measured utilizing a shortened version of the geriatric depression scale. Physical function (yes, no) was evaluated utilizing activities of daily living (ADL) and instrumental ADL.

### Statistical Analysis

To compare differences in participants’ general characteristics, chi-square tests were conducted, and the results were presented in frequencies and percentages. To explore the association between the utilization of senior centers and participation in health check-ups, logistic regression analysis was performed. Furthermore, the frequency of senior center utilization (five times or more per week, 3–4 times, 1–2 times, zero times) and the family composition of senior center users (living with family, living alone) were each subdivided into subgroups. The logistic regression analysis, indicated with a 95% confidence interval (CI) and odds ratio (OR), was conducted utilizing SAS version 9.4. Statistical significance for all analyses was set at a p-value of less than 0.05.

## Results

Table 1 presents the differences in the general characteristics of the population stratified by gender. The participation rate in health check-ups was 39.4% (N = 1590) for male participants and 42.2% (N = 2561) for female participants, indicating that women were more inclined to participate in health check-ups than men. The likelihood of participation increased with age, lower nutritional status, non-alcohol consumption, physical impairment, lack of physical activity, and self-perceived poor health.

**Table 1:** General characteristics of the study population by gender.

Variables	Male					Female				
	Check-ups		Non-checkups		p-value	Check-ups		Non-checkups		p-value
	N	(%)	N	(%)		N	(%)	N	(%)	
Total (N = 10,097)	1590	(39.4)	2445	(60.6)		2561	(42.2)	3501	(57.8)	
Senior center										
Use	604	(49.3)	621	(50.7)	< 0.0001	1244	(47.4)	1380	(52.6)	< 0.0001
Not use	986	(35.1)	1824	(64.9)		1317	(38.3)	2121	(61.7)	
Age										
65–69	458	(31.6)	993	(68.4)	< 0.0001	676	(32.7)	1394	(67.3)	< 0.0001
70–79	819	(43.6)	1061	(56.4)		1203	(46.3)	1397	(53.7)	
≥ 80	313	(44.5)	391	(55.5)		682	(49.0)	710	(51.0)	
Education										
≤ Middle school	917	(42.3)	1249	(57.7)	0.0002	2123	(44.7)	2628	(55.3)	< 0.0001
High school	538	(36.4)	940	(63.6)		390	(32.8)	800	(67.2)	
≥ College	135	(34.5)	256	(65.5)		48	(39.7)	73	(60.3)	
Job status										
Yes	737	(37.9)	1207	(62.1)	0.0612	805	(43.7)	1036	(56.3)	0.1235
No	853	(40.8)	1238	(59.2)		1756	(41.6)	2465	(58.4)	
Income										
High	731	(38.7)	1156	(61.3)	0.0953	883	(41.5)	1246	(58.5)	0.6293
Middle	483	(42.0)	668	(58.0)		624	(42.3)	851	(57.7)	
Low	376	(37.7)	621	(62.3)		1054	(42.9)	1404	(57.1)	
Family interaction										
High	242	(38.1)	393	(61.9)	0.0852	477	(44.3)	600	(55.7)	0.2287
Middle	979	(38.6)	1557	(61.4)		1571	(41.5)	2215	(58.5)	
Low	343	(42.8)	459	(57.2)		482	(43.0)	640	(57.0)	
Nutrition status										
High	10,319	(16.2)	53,439	(83.8)	< 0.0001	1601	(38.9)	2516	(61.1)	< 0.0001
Middle	3579	(24.0)	11,348	(76.0)		605	(47.5)	670	(52.5)	
Low	3580	(24.0)	11,349	(76.0)		355	(53.0)	315	(47.0)	
Alcohol status										
No	704	(42.1)	970	(57.9)	0.0037	2080	(44.0)	2645	(56.0)	< 0.0001
Yes	886	(37.5)	1475	(62.5)		481	(36.0)	856	(64.0)	
Smoking status										
No	1235	(40.3)	1831	(59.7)	0.0430	2488	(42.0)	3439	(58.0)	0.0049
Yes	355	(36.6)	614	(63.4)		73	(54.1)	62	(45.9)	
Physical function										
Poor	149	(46.9)	169	(53.1)	0.0046	300	(52.0)	277	(48.0)	< 0.0001
Good	1441	(38.8)	2276	(61.2)		2261	(41.2)	3224	(58.8)	
Physical activity										
No	926	(41.5)	1307	(58.5)	0.0028	1382	(45.9)	1627	(54.1)	< 0.0001
Yes	664	(36.8)	1138	(63.2)		1179	(38.6)	1874	(61.4)	
Self-reported health status										
High	868	(38.2)	1402	(61.8)	0.2335	1088	(40.7)	1582	(59.3)	0.0365
Middle	470	(40.6)	688	(59.4)		840	(42.8)	1122	(57.2)	
Low	225	(41.4)	318	(58.6)		592	(45.0)	725	(55.0)	
Depression										
Normal	1297	(39.5)	1983	(60.5)	0.3937	1904	(41.8)	2651	(58.2)	0.0063
Moderate	204	(37.4)	342	(62.6)		445	(41.2)	634	(58.8)	
Severe	89	(42.6)	120	(57.4)		212	(49.5)	216	(50.5)	

Table 2 presents the outcomes of the logistic regression analysis examining the correlation between the utilization of senior centers and participation in health check-ups. Among individuals utilizing senior centers, men and women were 1.69 and 1.23 times more likely to participate in health check-ups, respectively, compared with individuals who did not use utilize senior centers (men: adjusted OR: 1.69; 95% confidence interval, 1.46–1.97 p-value: < 0.0001; women: aOR, 1.23; 95% CI 1.10–1.39, p-value: 0.0002). Additionally, individuals with good physical function had lower odds of undergoing health check-up compared to those with poor physical function. (men: aOR: 0.70; 95% CI 0.53–0.94, p-value: 0.0169; women: aOR, 0.71; 95% CI 0.58–0.89, p-value: 0.0023).

**Table 2:** Results of logistic regression analysis investigating the association between senior center utilization and participation in health check-up.

Variables	Male		Female	
	aOR	95% CI	aOR	95% CI
Senior center				
Not use (ref)	1.00		1.00	
Use	**1.69**	**(1.46–1.97)**	**1.23**	**(1.10–1.39)**
Age				
65–69 (ref)	1.00		1.00	
70–79	**1.55**	**(1.32–1.82)**	**1.64**	**(1.43–1.89)**
≥ 80	**1.51**	**(1.21–1.90)**	**1.89**	**(1.58–2.26)**
Education				
≤ Middle school (ref)	1.00		1.00	
High school	0.89	(0.77–1.04)	0.73	(0.63–0.86)
≥ College	0.73	(0.57–0.94)	0.88	(0.60–1.31)
Job status				
No (ref)	1.00		1.00	
Yes	1.02	(0.88–1.19)	**1.29**	**(1.15–1.47)**
Income				
Low (ref)	1.00		1.00	
Middle	**1.44**	**(1.20–1.74)**	**1.23**	**(1.08–1.41)**
High	**1.26**	**(1.05–1.53)**	1.09	(0.95–1.26)
Family interaction				
Low (ref)	1.00		1.00	
Middle	0.86	(0.69–1.08)	**1.21**	**(1.02–1.45)**
High	0.89	(0.75–1.06)	1.11	(0.96–1.28)
Nutrition status				
High (ref)	1.00		1.00	
Middle	**1.41**	**(1.18–1.69)**	**1.35**	**(1.18–1.56)**
Low	**2.60**	**(1.95–3.47)**	**1.69**	**(1.40–2.05)**
Alcohol status				
No (ref)	1.00		1.00	
Yes	0.90	(0.78–1.04)	0.77	(0.67–0.88)
Smoking status				
No (ref)	1.00		1.00	
Yes	0.91	(0.78–1.08)	**1.67**	**(1.17–2.39)**
Physical function				
Poor (ref)	1.00		1.00	
Good	0.70	(0.53–0.94)	0.71	(0.58–0.89)
Physical activity				
No (ref)	1.00		1.00	
Yes	**1.25**	**(1.09–1.44)**	**1.5**	**(1.35–1.68)**
Self-reported health status				
High (ref)	1.00		1.00	
Middle	0.93	(0.80–1.10)	0.93	(0.82–1.06)
Low	0.88	(0.70–1.12)	0.83	(0.71–0.98)
Depression				
Normal (ref)	1.00		1.00	
Moderate	0.69	(0.57–0.86)	0.83	(0.72–0.97)
Severe	0.74	(0.53–1.05)	1.09	(0.87–1.38)

Significant values are given in bold.

*aOR* adjusted odds ratio, *CI* confidence interval.

Table 3 presents the subgroup analysis of health check-up participation according to the utilization of senior centers. Among women with severe depression, those utilizing senior centers were 1.59 times more likely to participate in health check-ups compared with those who did not utilize these centers (aOR, 1.59; 95% CI 1.03–2.46, p-value: < 0.0001).

**Table 3:** Subgroup analysis of health check-up participation according to the use of elderly facilities.

Variables	Male			Female		
	Not use	Use		Not use	Use	
		aOR	95% CI		aOR	95% CI
Age						
65–69	1.00	2.29	(1.68–3.14)	1.00	1.49	(1.20–1.86)
70–79	1.00	1.65	(1.35–2.02)	1.00	1.26	(1.08–1.49)
≥ 80	1.00	1.32	(0.96–1.84)	1.00	0.88	(0.70–1.12)
Education						
≤ Middle school	1.00	1.64	(1.36–1.99)	1.00	1.12	(1.00–1.28)
High school	1.00	1.67	(1.26–2.21)	1.00	1.75	(1.32–2.35)
≥ College	1.00	1.59	(0.94–2.70)	1.00	2.50	(0.80–7.82)
Job status						
Yes	1.00	2.41	(1.92–3.03)	1.00	1.43	(1.15–1.78)
No	1.00	1.22	(1.00–1.50)	1.00	1.15	(1.01–1.33)
Income						
Low	1.00	2.27	(1.78–2.91)	1.00	1.63	(1.33–2.00)
Middle	1.00	1.40	(1.08–1.83)	1.00	1.25	(1.00–1.58)
High	1.00	1.44	(1.07–1.94)	1.00	1.00	(0.84–1.20)
Family interaction						
Low	1.00	2.31	(1.56–3.42)	1.00	1.84	(1.40–2.43)
Middle	1.00	1.79	(1.49–2.17)	1.00	1.27	(1.10–1.47)
High	1.00	1.33	(0.95–1.86)	1.00	0.90	(0.69–1.18)
Nutrition status						
High	1.00	1.97	(1.65–2.36)	1.00	1.34	(1.17–1.55)
Middle	1.00	1.31	(0.94–1.84)	1.00	0.99	(0.78–1.27)
Low	1.00	1.11	(0.61–2.03)	1.00	1.37	(0.97–1.96)
Alcohol status						
No	1.00	1.55	(1.23–1.96)	1.00	1.09	(0.97–1.25)
Yes	1.00	1.89	(1.56–2.32)	1.00	2.10	(1.62–2.74)
Smoking status						
No	1.00	1.63	(1.38–1.94)	1.00	1.22	(1.10–1.38)
Yes	1.00	2.12	(1.54–2.93)	1.00	1.72	(0.62–4.76)
Physical function						
No	1.00	1.71	(1.47–2.01)	1.00	1.22	(1.09–1.38)
Yes	1.00	1.54	(0.89–2.69)	1.00	1.15	(0.76–1.74)
Physical activity						
No	1.00	1.65	(1.36–2.02)	1.00	1.26	(1.08–1.48)
Yes	1.00	1.79	(1.43–2.25)	1.00	1.24	(1.05–1.47)
Self-reported health status						
High	1.00	2.10	(1.72–2.59)	1.00	1.61	(1.35–1.92)
Middle	1.00	1.42	(1.09–1.86)	1.00	0.96	(0.79–1.18)
Low	1.00	1.11	(0.74–1.67)	1.00	1.11	(0.88–1.41)
Depression						
Normal	1.00	1.94	(1.64–2.29)	1.00	1.32	(1.16–1.51)
Moderate	1.00	1.07	(0.70–1.66)	1.00	0.93	(0.71–1.23)
Severe	1.00	0.73	(0.37–1.49)	1.00	1.59	(1.03–2.46)

*aOR* adjusted odds ratio, *CI* confidence interval.

Additionally, subgroup analyses stratified by senior center utilization frequency and user’s family composition were conducted. As exhibited in Table 4, both sexes display a progressively increasing likelihood of participating in health check-ups as the frequency of utilizing senior centers per week rises. Male and female participants who utilized senior centers 1–2 times per week had 1.55-fold (95% CI 1.28–1.90) and 1.29-fold (95% CI 1.11–1.50,) higher odds, respectively, of participating in health check-ups compared with those who did not utilize these senior centers. Male and female participants utilizing the senior center 3–4 times had 1.64-fold (95% CI 1.34–2.02,) and 1.29-fold (95% CI 1.11–1.50 higher odds, respectively, and those using them five times or more had 2.47-fold (95% CI 1.97–3.12) and 1.84-fold (95% CI 1.59–2.15) higher odds, respectively, compared with those not utilizing senior centers.

**Table 4:** Results of subgroup analysis stratified by frequency of use of facilities for the elderly and family composition of users.

Variables	Male		Female	
	aOR	95% CI	aOR	95% CI
Frequency of visits to senior center				
0 (ref)	1.00		1.00	
1–2	1.55	(1.28–1.90)	1.29	(1.11–1.50)
3–4	1.64	(1.34–2.02)	1.29	(1.11–1.50)
5-	2.47	(1.97–3.12)	1.84	(1.59–2.15)
Senior center				
Not use (ref)	1.00		1.00	
Use				
Living with family	1.72	(1.49–2.00)	1.39	(1.23–1.58)
Living without family	2.11	(1.63–2.73)	1.52	(1.34–1.73)

*aOR* adjusted odds ratio, *CI* confidence interval.

The analysis was conducted by classifying individuals who use the senior citizen center based on whether they live with their families. Compared to those who do not use the senior citizen center, it was found that elderly individuals living alone are more likely to receive health check-ups when utilizing the senior citizen center (men: aOR, 2.11; 95% CI 1.63–2.73; women: aOR, 1.52; 95% CI 1.34–1.73). All p-values for the subgroup analysis were < 0.0001.

## Discussion

This study identified an association between the utilization of senior centers and participation in health check-ups. Individuals utilizing senior centers exhibited a higher inclination of participating in health check-ups compared with those who did not utilize these facilities. This finding, controlling for family interaction, suggests that interactions among peers excluding family members, might influence participation in health check-ups among older adults. Moreover, there was a progressive increase in the likelihood of participating in health check-ups as the frequency of utilizing senior centers increased. Among users of senior centers, individuals living alone exhibited a higher likelihood of participating in health check-ups. This underscores the potential association between social relationships and participation in health check-ups.

It well-established that social relationships play various positive roles in the lives of older adults, encompassing health and more^39^. Previous studies have emphasized the significant role of social networks in promoting various health-related behaviors and facilitating health check-ups^40,41^. Other research has demonstrated that older adults with close family relationships and active engagement in social activities are more likely to undergo regular health check-ups^42,43^. The greater the social support from family, friends, and others, the stronger the association with participation in health check-ups^44^. Furthermore, it has been noted that this association extends not only to health check-ups but also to preventive health behaviors such as vaccinations^23^. Previous studies have indicated the association between social support and participation in health check-ups for both men and women^31^. This association was also found to be significant for both genders in the current study. Additionally, it demonstrated that participants engaging in social activities, such as utilizing senior centers, were more likely to participate in health check-ups than those who did not. However, unlike previous studies, this research focused on interactions with peers through the utilization of senior centers, controlling for interactions with family and relatives. Moreover, the analysis of the relationship between usage frequency and participation in health check-ups indicated a correlation between increased social activity and a higher likelihood of participating in health check-ups.

According to Andersen’s healthcare utilization model, healthcare utilization is influenced by four elements: environmental, demographic characteristics, health behavior, and health outcomes^22^. From a psychological perspective, social isolation in old age is considered a potential obstacle to participation in health programs^21,45^. Socially isolated individuals often place lower value on health compared with their non-isolated counterparts^24^. This diminished valuation of health acts as a deterrent to engaging in health management behaviors^24^. Socialization and social support serve as motivational factors for older adults to consistently engage in health behaviors^25^. Previous research has highlighted that, while individuals recognize the importance of improving health through health behaviors, they might naturally engage in health-promoting activities as part of their involvement in social activities^25^. Among older adults, self-efficacy promotes health-seeking behaviors and becomes a predictive concept for actions such as health check-ups^46^. Older adults with higher self-efficacy are more likely to utilize preventive medical care and undergo health check-ups compared with those with lower self-efficacy^47^. Additionally, friendship interactions among older adults exhibit a positive correlation with self-efficacy^48^.

This study has several limitations. First, it is a cross-sectional study, which hinders the determination of causality between factors, making it challenging to evaluate the sequence of events leading to outcomes. Second, the reliance on self-reported survey methods might compromise the reliability of the results. Third, despite attempts to control for covariates that may influence the dependent variable, uncontrolled confounding variables might have impacted the results. Fourth, variables related to hospitalization or long-term care admission could not be controlled for, which may have influenced the results.

However, this study also possesses several strengths. First, the data utilized in the study targeted the national population, enhancing its representativeness. Second, conducting face-to-face interviews by trained professionals could enhance the reliability and validity of survey responses.

## Conclusion

The results of this study suggest a potential association between the use of community centers by the elderly and participation in health screenings. With the global population aging, the efficient management of older adult health has become a critical area of interest. This study suggests the possibility that facilities where the elderly gather could be utilized for health management purposes.

## Data Availability

The data can be accessed through a link provided to public data. https://chs.kdca.go.kr/chs/index.do.
